# Multifactorial Analysis of the Effect of Applied Gamma-Polyglutamic Acid on Soil Infiltration Characteristics

**DOI:** 10.3390/polym16202890

**Published:** 2024-10-14

**Authors:** Shikai Gao, Xiaoyuan Zhang, Songlin Wang, Yuliang Fu, Weiheng Li, Yuanzhi Dong, Yanbin Li, Zhiguang Dai

**Affiliations:** 1 School of Water Conservancy, North China University of Water Resources and Hydropower, Zhengzhou 450045, China; igaoshikai@163.com (S.G.); z202210010114@stu.ncwu.edu.cn (X.Z.); wangsonglin@ncwu.edu.cn (S.W.); 16634868315@163.com (W.L.); dongyuanzhi0@126.com (Y.D.); liyb101@163.com (Y.L.); 2 College of Agricultural Engineering, Henan University of Science and Technology, Luoyang 471003, China; daizhiguang100@163.com

**Keywords:** γ-polyglutamic acid, infiltration characteristics, Hydrus-1D, capacitor charging

## Abstract

To investigate the mechanism and influence of applying gamma-polyglutamic acid (γ-PGA) on soil water infiltration, laboratory experiments and numerical simulations were conducted using Hydrus-1D. These studies assessed the impact of various application rates of γ-PGA on soil water characteristic parameters. Orthogonal simulation experiments on soil bulk density, γ-PGA application rates, and burial depths were performed utilizing predefined soil water characteristic values (twelve groups: nine groups of numerical simulation experiments and three groups of laboratory verification tests), and the soil infiltration characteristics were analyzed. Concurrently, an empirical model was developed to elucidate the relationships between the empirical model parameters and influencing factors, as well as to examine the sensitivity of these factors to changes in soil infiltration rate. The relationship between cumulative infiltration and the distance of wetting front movement, based on the water balance equation, was refined. The results indicated that γ-PGA significantly affected soil water characteristic parameters, where the saturated water content and the reciprocal of soil intake suction increased with rising γ-PGA applications (*p* < 0.01), while the saturated hydraulic conductivity and the parameter n decreased (*p* < 0.01), with no notable changes in the retained water content (*p* > 0.05). The trend in cumulative infiltration influenced by various factors could be modeled by a capacitive charging model function, which yielded a superior fit. A negative correlation existed between the sensitivity index and all the influencing factors (*p* < 0.05), with the order of influence being soil bulk density, γ-PGA application rate, and γ-PGA burial depth, respectively. Utilizing the modified water balance equation, the ratio of cumulative infiltration to wetting front migration distance corresponded more closely with a power function. These findings provide a theoretical foundation for further studies on the effects of γ-PGA on crop growth characteristics in fields and the optimization of γ-PGA technical element combinations.

## 1. Introduction

γ-Polyglutamic acid (γ-PGA) was first identified in the outer capsule of Bacillus anthracis by Ivanovics in 1933 as a water-retaining agent [1]. As an anionic natural polymer synthesized by microorganisms, γ-PGA boasts excellent water solubility, superb adsorption, non-toxicity, and complete biodegradability by microorganisms [2]. It is also readily degraded by various hydrolases in the natural environment [1]. Moreover, due to its strong adsorption, chelation, freeze resistance, and flocculation properties for metal cations [3], it is particularly useful in improving soil quality, controlling soil water pollution, enhancing the effectiveness of chemical fertilizers and pesticides, and remediating soil pollution [1,2,4].

Currently, scholars globally have undertaken preliminary research on γ-PGA in agriculture and soil enhancement. Studies indicate that γ-PGA, when combined with soil, promotes the formation of soil aggregates and prevents soil erosion [5]. It also exhibits a robust acid–base buffering capacity, effectively stabilizing soil pH and preventing soil acidification and compaction from prolonged chemical fertilizer use [6]. Further research demonstrates that γ-PGA can enhance soil permeability, improve nitrogen fertilizer uptake by plant roots, and boost fertilizer efficiency [7]. Findings show that γ-PGA significantly increases the initial water-holding porosity, maximum water-holding capacity, and levels of ammonium nitrogen, nitrate nitrogen, available phosphorus, available potassium, exchangeable magnesium, and soil electrical conductivity while reducing aeration porosity and pH [8]. Additionally, γ-PGA enhances the effective water content and reduces the proportion of ineffective and excess water, particularly in chalky loam compared to sandy loam. In terms of soil water infiltration and holding capacity, γ-PGA effectively elevates the water-holding capacity, increases the saturated water content (*θs*), and the inverse value of inlet suction (α) while decreasing the n-value, which represents the rate of water loss [9,10]. It has been observed that γ-PGA reduces soil permeability and saturated hydraulic conductivity but does not alter the suction at the wetting front [11]. Its application increases soil saturated and surface water content while reducing deeper soil water content, thereby maintaining water within the intended wetting horizon, and reducing deep percolation. This leads to higher soil moisture levels and improved water use efficiency for crops and irrigation [12,13]. Additionally, γ-PGA modifies the water distribution within the soil profile, concentrating moisture in the root zone, which enhances crop water use efficiency [14]. In terms of γ-PGA’s impact on soil structure, nutrient balance, and crop yield, it has been noted that the volume of water-holding gel significantly swells and is more likely to increase soil expansion when soil capacity is low [8]. Additionally, γ-PGA, as an environmentally friendly and biodegradable polymer, can enhance soil structural stability, increase organic matter content, and boost enzyme activities such as urease, sucrase, and alkaline phosphatase. It promotes soil microbial activity and contributes to overall soil health improvement. Moreover, γ-PGA reduces soil permeability, thereby lowering the risk of agricultural chemical leaching and decreasing the likelihood of groundwater contamination [15]. The application of γ-PGA also improves nutrient uptake efficiency and drought resistance in crops while enhancing soil structure and aeration [13]. These studies indicate that the use of γ-PGA in agriculture not only helps to enhance soil fertility and crop yield but also protects the environment by reducing the chemical pollution of water resources.

In conclusion, while extensive research has explored the impact of γ-PGA on crop growth characteristics, studies examining its effects on soil infiltration capacity and texture are far more limited, often qualitative, and generally focused on single-factor analyses. Quantitative studies investigating infiltration characteristics under multifactorial influences are scarce, and the existing research often lacks broader applicability. Many model parameters lack well-defined physical interpretations, hampering the understanding of the internal mechanisms that regulate soil water infiltration. Thus, building on previous research regarding the effects of γ-PGA on crop growth, this study innovatively examines the multifactorial impact of three critical variables—soil bulk density, application rate, and burial depth—on soil water infiltration characteristics through a combination of laboratory experiments and numerical simulations utilizing the Hydrus-1D software version 4.17.0140. The orthogonal simulation experiment design employed in this study enabled a systematic evaluation of multiple variables, enhancing both the efficiency and scientific rigor of the research. Furthermore, the empirical model developed in this study not only incorporates physically meaningful parameters but also provides clarity, offering new insights into the influence of γ-PGA on soil water movement. A sensitivity analysis further identified the key factors affecting soil infiltration rates, offering precise guidance for the application of γ-PGA. These innovations not only enrich theoretical research but also provide a solid foundation and practical guidance for the application of γ-PGA in agricultural soil management, offering new strategies and approaches for sustainable agricultural development.

## 2. Materials and Methods

### 2.1. Experimental Materials

#### 2.1.1. The Soil Sample

The test soil sample is classified as sandy loam according to the United States Department of Agriculture (USDA) standards. This sandy loam was collected from the farming layer (0–20 cm) of Xiamaguan County, Wuzhong City, Ningxia. After collection, the soil was naturally dried, crushed, and sieved through a 2.0 mm standard sieve. The particle size distribution of the soil samples was analyzed using a Mastersize-2000 laser particle size analyze from Malvern, UK employing a Hy-dro2000SU large-capacity wet dispersion system. The soil particle grading composition and basic physical and chemical properties are detailed in Table 1.

#### 2.1.2. Source of the γ-PGA for the Test

The test γ-PGA was supplied by Shandong Furida Biotechnology Co., Ltd. (Jinan, China). It is a white, non-toxic, and tasteless powder with a particle size of 100 meshes and a molecular weight of 700 kD. To prepare, 2 g of γ-PGA was placed in a dialysis bag, and the total mass of the γ-PGA and the dialysis bag was weighed. Both ends of the dialysis bag were sealed and submerged in a beaker containing 1000 mL of distilled water. After soaking for 12 h, the dialysis bag was removed and hung in the air for 15 min until no water dripped from the bag. The mass of the dialysis bag was then weighed. The water absorption multiple of γ-PGA was calculated using the following formula:

(1) $$A=\frac{m_{3}-m_{2}}{m_{2}-m_{1}}$$

In the formula, A—water absorption multiple; $m_{1}$—quality of the dialysis bag, g; $m_{2}$—total mass of γ-PGA and the dialysis bag, g; and $m_{3}$—the weight measured after soaking for 12 h and air-drying for 15 min, g.

Each experimental group was repeated three times, and the average water absorption multiple of the γ-PGA in distilled water was determined to be 731 g/g.

### 2.2. Test Method

#### 2.2.1. Physical and Chemical Properties of γ-PGA

**Figure 1:**
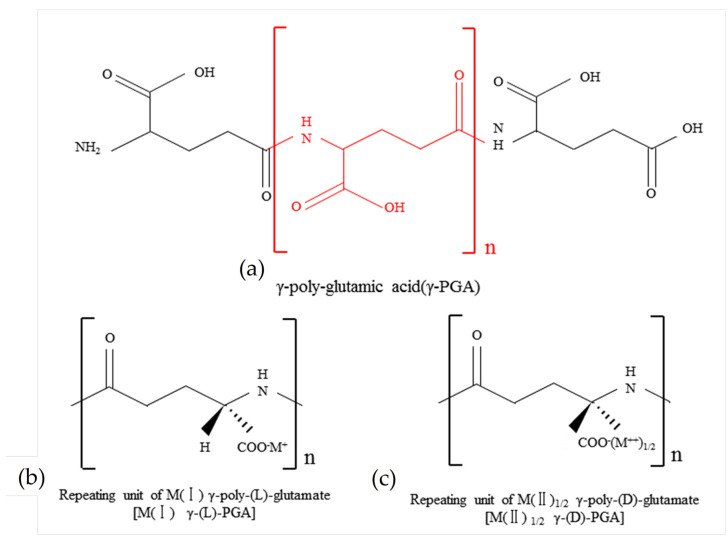
Molecular structure formula of γ-PGA.

γ-PGA is a polypeptide molecule comprising d-glutamic acid (d-Glu) and l-glutamic acid (l-Glu) monomers which are linked by an amide bond between the α-amino and γ-carboxyl groups. The molecular structure is illustrated in Figure 1. γ-PGA, sourced from FREDA Biotechnology Co., Ltd. (Jinan, China), is a non-toxic, water-soluble, biodegradable white powder with a molecular weight of 700 kD and a degradation period of up to two years in the soil. In the laboratory tests, 2 g of γ-PGA was placed in a dialysis bag ($m_{1}$), and the total mass of the bag and additive was recorded as $m_{2}$. After sealing and immersing the bag in 1000 mL of distilled water for 12 h, it was suspended for 15 min to allow dripping to cease, and the final mass was recorded as $m_{3}$. The water absorption capacity is calculated using the formula ($m_{3}-m_{2}$)/($m_{2}-m_{1}$). This test was conducted three times for each sample group, yielding average water absorption multipliers of 731 g/g for distilled water and 319 g/g for tap water.

#### 2.2.2. Indoor Test Materials and Devices

The indoor experiment was carried out at the State Key Laboratory of Ecological Water Conservancy in the Northwest Dry Region, Xi’an University of Technology, from 2016 to 2018.

**Figure 2:**
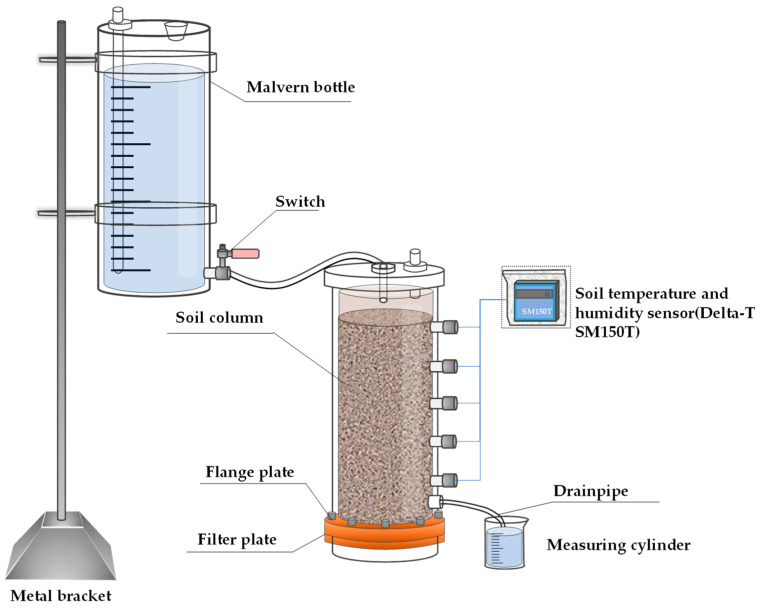
The experimental apparatus of one-dimensional vertical infiltration.

The apparatus primarily consists of a soil column and a Markov bottle as shown in Figure 2. The soil column is available in two sizes: 48 cm in height with a 9 cm inner diameter and 10 mm thickness, and 100 cm in height with an 18 cm inner diameter and 10 mm thickness. The Markov bottle used in the vertical water infiltration tests measures 50 cm in height, 5 cm in diameter, and 10 mm in thickness. The borrow holes are strategically placed in a vertical quincunx arrangement along the soil column, with each hole measuring 2.0 cm in diameter and spaced 5.0 cm apart. Additionally, to facilitate free drainage and minimize air resistance impact, exhaust holes are positioned at the bottom of the soil column.

#### 2.2.3. Test Protocol and Analysis Method

1. Indoor test

Utilizing a one-dimensional vertical infiltration device, a soil water infiltration test was conducted to assess the effects of γ-PGA application.

Study Design 1: To accurately predict the impact of γ-polyglutamate on soil water characteristics, a combination of indoor testing and numerical modeling was employed. γ-PGA was mixed with the screened soil samples at the concentrations of 0.10%, 0.20%, and 0.30% (mass ratio) corresponding to the bulk densities of 1.30 g/cm^3^, 1.35 g/cm^3^, and 1.40 g/cm^3^, respectively. The mixture was layered into soil columns (48 cm height, 9 cm inner diameter, and 10 mm thickness) up to a soil depth of 45 cm. The top 3 cm of the column was reserved for a water supply bottle (50 cm height, 5 cm inner diameter, and 10 mm thickness). A filter paper was placed on the surface of the soil sample to ensure uniform infiltration, with a water head of 2.0 cm. The experiment included four treatment groups: 0.10%, 0.20%, and 0.30% γ-PGA applications, and an untreated control group.

Study Design 2: Building on the results from Test Study Design 1, a multifactorial one-dimensional vertical water infiltration simulation was conducted to study the effects of soil bulk density (ρ), γ-PGA concentration, and application depth on the soil moisture and infiltration characteristics. The orthogonal test design comprised twelve groups, including nine numerical simulations and three indoor verification tests. The verification group used soil columns measuring 100 cm in height, 18 cm in diameter, and 10 mm in thickness, filled to a depth of 95 cm. The top 5 cm of the column was reserved for water supply (50 cm height, 5 cm inner diameter, and 10 mm thickness). A filter paper covered the soil sample surface to maintain consistent infiltration conditions, with a water seepage head of 2.50 cm. The specific parameters are outlined in Table 2.

2. Numerical model establishment
(1) Governing equation of flow movement

Assuming the soil is homogeneous and isotropic, with the indoor test control room temperature maintained at 20 °C and no air resistance, and disregarding source–sink terms such as root water absorption, the one-dimensional Richards equation for a variable saturated isotropic rigid porous medium, with water content as the dependent variable, is presented below:

(2) $$\left[D(\theta )\frac{\partial \theta }{\partial z}\right]+\frac{\partial K(\theta )}{\partial z}$$

(3) $$D(\theta )=K(\theta )\frac{\partial K(\varphi_{m})}{\partial z}$$

In these equations, θ represents the volumetric water content, cm^3^/cm^3^. K(θ) denotes the unsaturated hydraulic conductivity (cm/min). D(θ) is the water diffusivity of unsaturated soil (cm^2^/min). z is the disposition in the vertical direction (cm), with the upward direction being positive, and *t* indicates the infiltration time (min).

According to the van Genuchten model, the relationships among the volumetric water content, matrix potential, and hydraulic conductivity are established.

(2) Initial conditions and the boundary conditions

Initial condition:

(4) $$\theta (z)=\theta_{0}(z),\;0\leq z\leq 45\;\mathrm{cm},\;t=0$$

Upper boundary condition:

(5) $$h(z,t)=h_{0}(z,t),\;z=0,\;0\leq t<t_{\mathrm{input}}$$

Lower boundary condition:

(6) $$\frac{\partial h}{\partial z}=0,\;z=45\;\mathrm{cm},\;0\leq t<t_{\mathrm{input}}$$

In these conditions, $\theta_{0}(z)$ denotes the initial volumetric moisture content (cm^3^/cm^3^), and $h_{0}(z,t)$ is the depth of water accumulation on the soil surface (2 cm).

(3) Simulation method
① Determination of water characteristic parameters

The RETC software version 6.02 was utilized to predict the soil moisture characteristic parameters based on sandy loam soil data from the USDA. The inputs included soil bulk density ρ, the saturated volume water content of soil treated with γ-PGA, and the saturated hydraulic conductivity Ks, along with water suction values at 33 kPa and 1500 kPa corresponding to the γ-PGA-treated soil. The Rosetta Lite v.1.1 inversion calculation module was used for simulation prediction, yielding results for residual moisture content *θr*, reciprocal intake suction α, soil pore distribution parameter n, and soil pore connectivity parameter l, both test at 0.5. Based on Study Design 1, these predicted water characteristic parameters, alongside the cumulative infiltration measured in the one-dimensional vertical in-filtration test, were input as initial values into the Hydrus-1D model to optimize the water characteristic parameters for γ-PGA-enhanced soil.

② Analyses of simulated trials

As per Study Design 2 (Table 2), treatments 1–9 were the simulation test treatments derived from the Hydrus-1D model calculations, while treatments 10–12 were for indoor verification. An empirical model was developed from treatments 1–9 to analyze the correlations between the water infiltration characteristics of the γ-PGA-treated soil, soil bulk density, γ-PGA application rate, and the depth of γ-PGA application.

3. Observation project

① Determination of cumulative infiltration: During irrigation, a stopwatch was used to time the infiltration of soil water over a period of 360 min. The decrease in the water level of the Markov bottle was recorded to determine the cumulative infiltration.

② Determination of wet front transport distance and soil volume moisture content: The wet front movement distance was marked on the sidewall of the soil column, and the profile soil moisture content was monitored by a METER device at 5 cm intervals (ranging from 5 to 40 cm below the soil surface).

4. Statistical analysis method.

Multiple comparison tests were conducted using the minimum significant difference method. An empirical model was established through a multiple regression analysis, and relative deviation values were used for a comparative analysis between the calculated and measured values.

## 3. Results

### 3.1. Study on Influencing Factors of Soil Infiltration Characteristics with γ-Polyglutamic Acid Applied

Based on Experimental Study Design 2, a comparison of the measured soil moisture parameters and optimized inversion values under varying conditions of bulk density and water-retaining agent content was performed. The results are presented in Table 3.

It is evident that both the residual soil moisture content (*θr*) and the saturated moisture content (*θs*) increase with the addition of γ-PGA, while they decrease as soil bulk density increases. The rise in the soil residual water content due to the increased γ-PGA content was insignificant, with no notable differences between treatments. At a soil bulk density of 1.30 g/cm^3^, the saturated water content increased by 3.15%, 6.78%, and 7.33% in soils with 0.10%, 0.20%, and 0.30% γ-PGA content, respectively, compared to the untreated soil; at 1.35 g/cm^3^, the increases of 3.29%, 6.89%, and 7.30% were observed; and at 1.40 g/cm^3^, the increases were 4.15%, 8.17%, and 10.77%. These results confirm that γ-PGA significantly enhances soil water-holding capacity. The variable α, which is the reciprocal of soil intake suction, decreases with increasing soil bulk density. This decrease indicates that higher soil bulk density enhances soil intake suction and resistance to water loss. Conversely, soil intake suction decreases with increasing γ-PGA content for the same bulk density. According to Fu Yuliang et al., an increase in the reciprocal of soil intake suction enhances soil effective porosity (both non-capillary and capillary) and reduces ineffective porosity [16]. This suggests that higher γ-PGA application rates facilitate the soil reaching a water loss state during dehydration. The n-value, representing the water loss rate on the soil moisture characteristic curve [17], increases with soil bulk density but decreases with added γ-PGA. The correlation coefficients (*R*^2^) of the soil moisture characteristic parameters predicted by Hydrus-1D inversion are all greater than 0.993. The values for the *ν_RMSE_* range from 0.001 to 0.024, for *ν_MAE_* from 0.001 to 0.017, and for *ν_ME_* from −0.0025 to 0.0019. The closeness of the *ν_RMSE_*, *ν_MAE_*, and *ν_ME_* values to zero demonstrates the effectiveness of the Hydrus-1D inversion in calculating moisture parameters for soils treated with γ-PGA.

### 3.2. Analysis of Soil Infiltration Characteristics with γ-Polyglutamic Acid Applied

#### 3.2.1. The Effect of Multifactor Changes on Cumulative Infiltration

**Figure 3:**
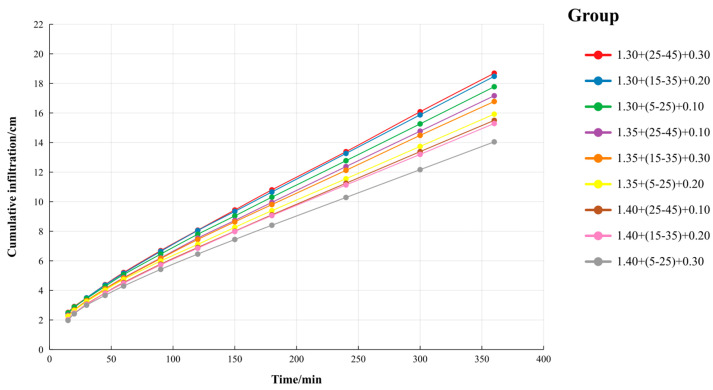
Cumulative infiltration under multifactors. Note: Each treatment is denoted as “a + b + c”, where “a” indicates the soil bulk density (g/cm^3^), “b” specifies the γ-PGA burial depth (cm), and “c” represents the γ-PGA application rate (mass percentage). For example, 1.30 + (25–45) + 0.30 indicates a treatment in which 0.30% γ-PGA (by mass) is applied at a burial depth of 25–45 cm in soil with a bulk density of 1.30 g/cm^3^.

Based on the moisture characteristic parameters detailed in Table 3, Hydrus-1D was utilized to simulate various treatments. Figure 3 illustrates the simulated values of cumulative infiltration for the soils treated with γ-PGA, influenced by multiple factors including soil bulk density, γ-PGA content, and application depth. Notably, Treatment 3 (with a bulk density of 1.30 g/cm^3^ + γ-PGA at a burial depth of 25~45 cm + 0.30% γ-PGA content) exhibited the largest cumulative infiltration amount. Conversely, Treatment 7 (a bulk density of 1.40 g/cm^3^ + γ-PGA at a burial depth of 5~25 cm + 0.30% γ-PGA content) had the smallest. This indicates that γ-PGA, being a super absorbent polymer, more effectively hinders the downward movement of soil water when applied shallowly rather than deeply, and this effect intensifies with increasing γ-PGA content. However, within the same soil bulk density, the burial depth and application rate of γ-PGA had no significant impact on cumulative infiltration. This suggests that soil bulk density and the method of γ-PGA application are crucial for its efficient use and broader adoption. Accordingly, the application rate of γ-PGA may be adjusted based on specific conditions to optimize agricultural economic benefits.

**Figure 4:**
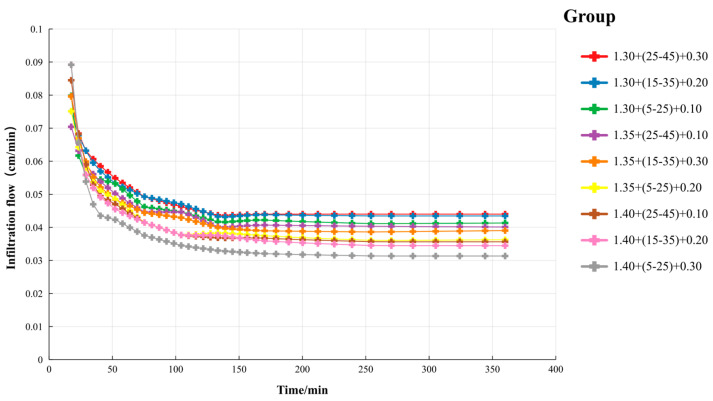
Infiltration flow under multifactor. Note: Each treatment is denoted as “a + b + c”, where “a” indicates the soil bulk density (g/cm^3^), “b” specifies the γ-PGA burial depth (cm), and “c” represents the γ-PGA application rate (mass percentage). For example, 1.30 + (25–45) + 0.30 indicates a treatment in which 0.30% γ-PGA (by mass) is applied at a burial depth of 25–45 cm in soil with a bulk density of 1.30 g/cm^3^.

The infiltration process diagram illustrates the dynamic changes in the soil water infiltration rate over time, influenced by the soil bulk density, the depth of γ-PGA application, and its dosage. The curves depicted in Figure 4 reveal two distinct stages of the infiltration process: an initial nonlinear stage followed by a linear stage. During the nonlinear stage, the infiltration rate drops rapidly, indicating that surface water quickly enters the soil and begins to saturate it. As time progresses, the infiltration rate stabilizes and transitions into the linear stage, where water primarily infiltrates through the soil’s capillary pores. In the early infiltration stage, the treatments with lower soil bulk density and higher γ-PGA dosage (e.g., 1.30 + (25–45) + 0.30) exhibit higher infiltration rates, suggesting that under these conditions, the soil absorbs water more rapidly. Over time, the infiltration rate in all the treatment groups shows a general declining trend, reflecting the gradual saturation of the soil.

Specifically, the infiltration rate in the 1.30 + (25–45) + 0.30 treatment group decreases significantly as time increases. This may be due to the water absorption effect of γ-PGA in the soil, which leads to rapid initial water uptake, but as water further infiltrates the soil, the rate begins to slow down. In contrast, the treatment groups with higher soil bulk density (e.g., 1.40 + (5–25) + 0.30) exhibit slower increases in infiltration rates over the same time period, indicating that higher soil bulk density limits the initial water infiltration speed. Figure 4 also reveals the impact of different γ-PGA application depths on the infiltration rate. The treatment groups where γ-PGA was applied in deeper soil layers (e.g., 1.35 + (25–45) + 0.10 and 1.40 + (25–45) + 0.10) show smaller declines in infiltration rate over the same period compared to those applied at shallower depths. This suggests that the application of γ-PGA in deeper soil layers helps maintain water infiltration for a longer duration. Additionally, the data points in the figure demonstrate the effect of γ-PGA dosage on infiltration rate. As the dosage increases, the soil’s infiltration rate rises rapidly during the initial stage, but this trend gradually slows over time, eventually converging with the other treatment groups. This may be because the hydrogel formed by γ-PGA in the soil increases the soil’s water retention capacity but also restricts further water infiltration.

In summary, the infiltration process diagram provides visual evidence of the effects of γ-PGA application on soil water infiltration rate, revealing the combined influence of the soil bulk density, γ-PGA dosage, and application depth on the dynamics of water infiltration.

To analyze the impact of each factor on cumulative soil infiltration, a multivariate analysis of variance using the least significant difference method was conducted between the factors and levels. The analysis, presented in Table 4, reveals that the soil bulk density and γ-PGA burial depth significantly affect soil cumulative infiltration (*p* < 0.01), and γ-PGA content also significantly influences this parameter (*p* < 0.01). In summary, the soil bulk density and γ-PGA application methods are pivotal for the judicious application of γ-PGA, with these findings contributing to reduced agricultural costs and promoting the environmentally sustainable development of agricultural soils.

#### 3.2.2. Calculation Model of Cumulative Infiltration

Further analysis suggests that the soil’s cumulative infiltration process resembles the capacitor charging process [16]. The model for cumulative water infiltration over time can be based on the capacitor charging model. It is assumed that there is a distinct transition between the two infiltration stages within this period. In the nonlinear infiltration stage, the cumulative infiltration amount, denoted as I1, follows the relationship derived from the capacitive charging model as shown below:

(7) $$I_{1}=A\cdot (1-e^{-\frac{t}{B}})$$

In the formula, $I_{1}$—the cumulative infiltration amount in the nonlinear stage (cm^3^/cm); A—volume constant, representing the maximum water infiltration limit in the nonlinear stage; and B—time constant, indicating the duration for water infiltration to transition from nonlinear to linear.

In the stable infiltration stage, the relationship of cumulative infiltration volume with time is expressed as follows:

(8) $$I_{2}=C\cdot t$$

Here, $I_{2}$—cumulative infiltration volume during the stable infiltration stage (cm^3^/cm) and C—soil infiltration rate in the stable infiltration stage (cm^3^/(cm·min)).

The total cumulative infiltration amount, I, for the entire infiltration process within the simulation timeframe is determined as follows:

(9) $$I=A\cdot (1-e^{-\frac{t}{B}})+C\cdot t$$

Based on the Hydrus-1D simulation calculations, the fitted parameters A, B, and C of the cumulative infiltration model under varying conditions of bulk density, content, and burial depth are presented. The results are displayed in Table 5.

According to the simulations using the Hydrus-1D software, it was found that the volume constant A, time constant B, steady infiltration rate C, soil bulk density *ρ*, γ-PGA application depth, and γ-PGA content are strongly related through a power exponential function. The relationship is formulated as follows:

(10) $$\left\{\begin{array}{l} A\left(\rho \begin{array}{c} \gamma \begin{array}{c} h \end{array} \end{array}\right)=\rho^{a}\cdot \gamma^{b}\cdot h^{c}\\ B\left(\rho \begin{array}{c} \gamma \begin{array}{c} h \end{array} \end{array}\right)=\rho^{a}\cdot \gamma^{b}\cdot h^{c}\\ C\left(\rho \begin{array}{c} \gamma \begin{array}{c} h \end{array} \end{array}\right)=\rho^{a}\cdot \gamma^{b}\cdot h^{c} \end{array}\right.$$

In the formula, A, B, and C, respectively, represent soil bulk density ρ, γ-PGA content γ, and γ-PGA application depth. h is the function value of the independent variable and a,b,andc represent the fitted exponential parameters.

A stepwise regression analysis method [18] was employed using MATLAB R2021b to establish a parameter fitting model. The quantitative relationships between the model coefficients A, B, and C variables such as soil bulk density ρ, γ-PGA application depth h, and γ-PGA content are as follows:

(11) $$\left\{\begin{array}{ll} A=\rho^{2.714}\cdot \gamma^{0.100}\cdot h^{0.105} & v_{RMSE}=0.089\;R^{2}=0.991\\ B=\rho^{7.907}\cdot \gamma^{0.181}\cdot h^{0.249} & v_{RMSE}=0.171\;R^{2}=0.996\\ C=\rho^{-10.034}\cdot \gamma^{-0.217}\cdot h^{-0.212} & v_{RMSE}=0.182\;R^{2}=0.981 \end{array}\right.$$

The coefficients of determination *R*^2^ are consistently above 0.98, and the relative deviation coefficient *ν_RMSE_* ranges from 0.089 to 0.182, indicating a strong fit for Formula (11).

Substituting Formula (11) into Formula (7) yields a quantitative calculation model for cumulative infiltration in relation to soil bulk density *ρ*, γ-PGA application depth *h*, and γ-PGA content γ under the influence of multiple factors, represented as follows:

(12) $$I=\rho^{2.714}\cdot \gamma^{0.100}\cdot h^{0.105}(1-e^{-\frac{t}{\rho^{7.907}\cdot \gamma^{0.181}\cdot h^{0.249}}})+\rho^{-10.034}\cdot \gamma^{-0.217}\cdot h^{-0.212}\cdot t$$

**Figure 5:**
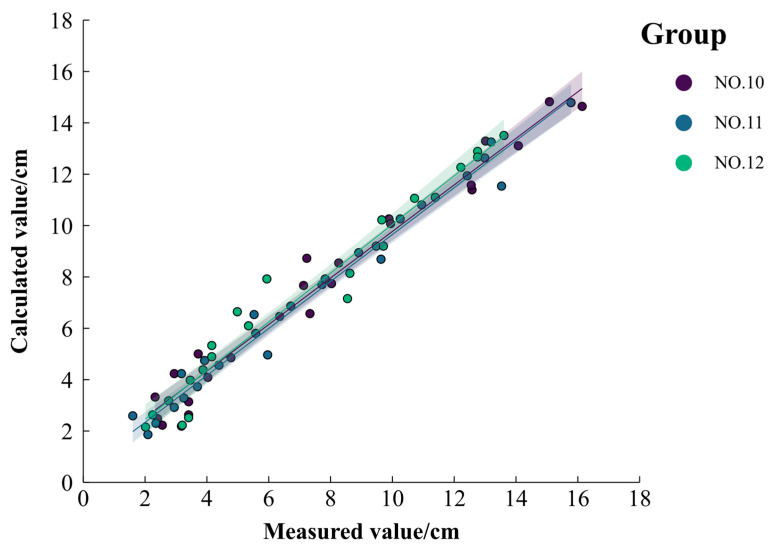
Comparison of measured and calculated values of cumulative infiltration.

To verify the reliability of this empirical model, the measured data from NO. 10, NO. 11, and NO. 12 were used. As shown in Figure 5, the scatter plot of the calculated versus measured values of cumulative infiltration generally aligns closely with the 1:1 diagonal line. The *ν_RMSE_* is 0.023 cm, and *R*^2^ is 0.983 (*p* < 0.01), demonstrating the model’s effectiveness in quantitatively describing the relationship between cumulative infiltration and various influencing factors.

**Figure 6:**
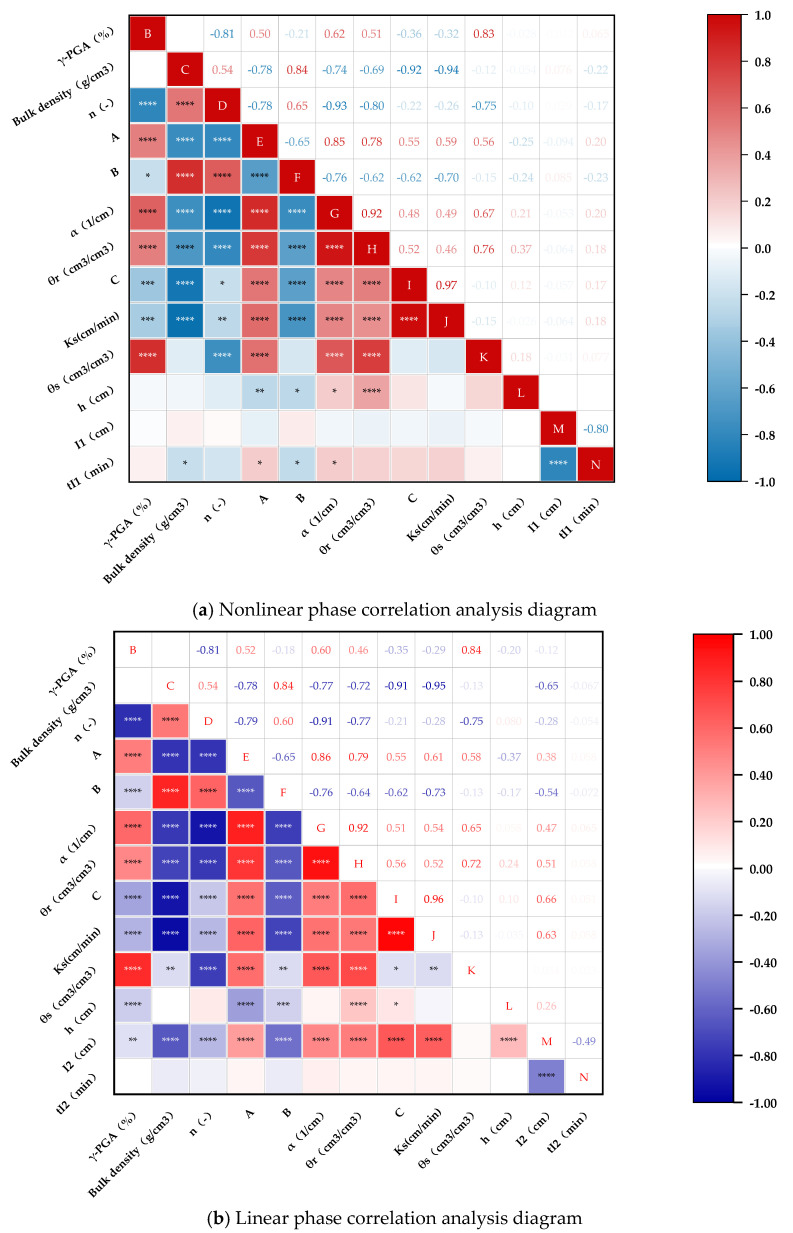
Correlation analysis plot between multifactors and model parameters. Note: * represents a weak statistical significance at the level (*p* ≤ 0.05); ** represents a higher statistical significance at the level (*p* ≤ 0.01); *** represents a very high statistical significance at the level (*p* ≤ 0.001); **** represents an extremely high statistical significance at the level (*p* ≤ 0.0001). The more asterisks, the greater the significance of the correlation, and smaller correlations are not highlighted. “B” represents γ-PGA (unit: %); “C” represents the bulk density (unit: g/cm^3^); “D” denotes the water loss rate (*n*); “E” refers to the volume constant (A); “F” indicates the time constant (B); “G” signifies the inverse inlet suction (α); “H” stands for the residual moisture content (*θr*) (unit: cm^3^/cm^3^); “I” denotes the soil infiltration rate during the stable infiltration stage (C) (unit: cm^3^/(cm·min)); “J” represents the saturated hydraulic conductivity (*Ks*) (unit: cm/min); “K” stands for the saturated moisture content (*θs*) (unit: cm^3^/cm^3^); “L” represents *h* (unit: g/cm^3^); “M” refers to *I*_1_ (unit: cm), and represents the cumulative infiltration amount in the nonlinear stage (cm^3^/cm) in (a); “M” refers to *I*_2_ (unit: cm), and represents the cumulative infiltration volume during the stable infiltration stage (cm^3^/cm) in (b); “N” indicates *tI*_1_ (unit: min) in (a); “N” also represents *tI*_2_ (unit: min) in (b).

To assess the significance of the simulation coefficients A, B, and C and factors such as soil bulk density *ρ*, γ-PGA content γ, γ-PGA application depth h, the Pearson coefficient method [19] was utilized to analyze their correlations. In order to clearly show the correlation between the cumulative penetration into the model parameters, the correlation analysis was divided into a nonlinear phase (Figure 5) and a linear stage (Figure 6) to analyze.

Overall, the influencing factors (bulk density ρ, γ-PGA content, and γ-PGA application depth *h*) exhibit distinct correlation effects with the fitting parameters: volume constant A, time constant B, stable permeability C, and infiltration volume *I*. A represents the maximum water infiltration limit during the nonlinear stage, while *B* indicates the duration for water infiltration to transition from the nonlinear stage (*tI*_1_) to the linear stage (*tI*_2_). *C* is the stable infiltration rate during the linear stage, and *I* represents the overall infiltration volume over time.

In terms of the effects of γ-PGA application depth *h* on infiltration characteristics, it was found that deeper γ-PGA application during the nonlinear stage *tI*_1_ is negatively correlated with the volume constant A (correlation coefficient = −0.54, *p* < 0.05), and the time constant B (correlation coefficient = −0.21, not significant). This suggests that while deeper application may slightly reduce the maximum infiltration volume and the duration of the infiltration process, these effects are not strongly pronounced. However, during the linear stage *tI*_2_, the depth of γ-PGA application *h* shows a significant positive correlation with the stable infiltration rate C (correlation coefficient = 0.52, *p* < 0.05), indicating that deeper application improves the soil’s ability to maintain consistent water flow over time. On the other hand, the infiltration volume *I* during the nonlinear stage *tI*_1_ is negatively correlated with the bulk density *ρ* (correlation coefficient = −0.75, *p* < 0.0001) and γ-PGA content *γ* (correlation coefficient = −0.81, *p* < 0.0001), indicating that higher bulk density and higher γ-PGA content reduce the initial infiltration capacity of the soil. This effect is likely due to the increased soil compaction and the formation of a denser hydrogel matrix, which restricts water movement during the early stages of infiltration. Conversely, during the linear stage *tI*_2_, the infiltration volume *I* shows a moderate positive correlation with γ-PGA application depth *h* (correlation coefficient = 0.52, *p* < 0.05), reinforcing the idea that the deeper application of γ-PGA enhances the soil’s long-term infiltration stability. These observations illustrate that while increasing the γ-PGA application depth can initially reduce infiltration rates during the nonlinear stage *tI*_1_, it ultimately benefits the soil’s ability to maintain consistent water infiltration during the linear stage *tI*_2_. Conversely, higher bulk density and γ-PGA content tend to inhibit water infiltration initially but have less impact during the stable infiltration phase. The nuanced interplay of these factors underscores the importance of carefully considering γ-PGA application strategies to optimize soil water management.

Regarding the effects of γ-PGA application depth (*h*) on infiltration characteristics, it was found that deeper γ-PGA application during the nonlinear stage *tI*_1_ reduces the overall infiltration volume *I* and shortens the duration of the infiltration process, leading to a quicker transition to the stable infiltration stage (*tI*_2_). This is likely due to the increased soil resistance to water movement as depth increases, which reduces the effectiveness of γ-PGA in enhancing soil moisture retention during the initial infiltration phase. Conversely, during the linear stage *tI*_2_, the deeper application of γ-PGA improves the stable infiltration rate C, suggesting that while initial water infiltration is restricted, the soil’s ability to maintain consistent water flow is enhanced over time.

The observed correlations demonstrate that a decrease in the bulk density ρ leads to looser soil with enhanced aeration, improving its capacity to store water and meet the water requirements of crops, particularly during the nonlinear stage *tI*_1_. This also significantly enhances the water storage ability of soil’s capillary pores. However, it substantially reduces the duration of water flow in the soil during the initial phase of infiltration, causing the soil to quickly reach a stable infiltration stage during the linear stage *tI*_2_, thereby increasing the water infiltration rate at this stage. Increasing the application of γ-PGA effectively boosts water storage in soil capillary pores due to its abundant hydrophilic carboxyl groups and peptide bonds that readily bond and absorb water, forming a hydrogel state. This alters the soil structure and porosity, thereby inhibiting deep water infiltration, especially during the nonlinear stage *tI*_1_. When applied in large amounts, γ-PGA forms a hydrogel state that quickly restricts water flow and the duration of water presence in the soil during the early infiltration phase *tI*_1_, yet it does not significantly impact the infiltration rate in the stable stage *tI*_2_.

In the correlation relationship diagram, the γ-PGA infiltration experiment was divided into two parts: the analysis of soil texture and physical properties during the nonlinear stage *tI*_1_, and the analysis of infiltration and water retention parameters during the linear stage *tI*_2_. Numerous studies have demonstrated that after applying γ-PGA in crop cultivation, it enhances the soil water-holding capacity, reduces the water loss rates [8,13], causes soil volume expansion to form aggregates [9], and effectively increases the soil moisture content and bulk density [10,12]. Additionally, the analysis of the results reveals a clear positive correlation between the saturated hydraulic conductivity *Ks* and the nonlinear infiltration parameters A and B during the nonlinear stage *tI*_1_, along with the constant term parameter C of the linear phase stable infiltration rate during the linear stage *tI*_2_. This suggests that as the soil hydraulic conductivity increases, the value of the stable infiltration rate C also increases, indicating effective infiltration stabilization performance during the linear stage *tI*_2_ [10]. The application rate of γ-PGA was positively correlated with the saturated water content *θs*, suggesting an improvement in soil infiltration and water retention capacity by enhancing soil structure. The experiments also demonstrated that applying γ-PGA could raise the residual water content *θr* and decrease intake suction α, thereby boosting the soil water-holding capacity [11]. However, the increase in water-holding capacity due to changes in the application rate was not significant, leading to a lack of correlation during both stages *tI*_1_ and *tI*_2_.

Increasing the γ-PGA application depth *h* is not conducive to improving the water storage capacity in soil capillary pores during the nonlinear stage *tI*_1_, and it shortens the duration of water infiltration from the nonlinear stage *tI*_1_ to the linear stage *tI*_2_, but it can improve the stable infiltration rate during the linear stage *tI*_2_.

#### 3.2.3. Dynamic Change in Soil Infiltration Rate

To determine the impact of various factors on the change in soil infiltration rate, Formula (12) is employed to derive the first derivative of cumulative infiltration per unit area with respect to time. This yields the functional relationship of multiple factors’ effects on soil infiltration rate over time when applying γ-polyglutamic acid:

(13) $$q=\frac{dI}{dt}=\rho^{-5.193}\cdot \gamma^{-0.081}\cdot h^{-0.144}e^{-\frac{t}{\rho^{7.907}\cdot \gamma^{0.181}\cdot h^{0.249}}}+\rho^{-10.034}\cdot \gamma^{-0.217}\cdot h^{-0.212}$$

In the formula, q represents the soil infiltration rate, expressed in cm/min, and the other symbols retain their meanings as defined previously.

As different factors affect the soil infiltration rate differently, the partial derivatives of these factors are calculated and their absolute values are taken to further analyze the sensitivity of each factor’s influence. For details, see Formulas (14) to (16).

(14) $$\left|\frac{dq}{d\rho }\right|=\left|\begin{array}{l} -5.193\rho^{-6.193}\cdot \gamma^{-0.081}\cdot h^{-0.144}e^{-\frac{t}{\rho^{7.907}\cdot \gamma^{0.181}\cdot h^{0.249}}}-7.907\rho^{-5.193}\cdot \gamma^{-0.081}\cdot h^{-0.144}\cdot e^{-\frac{t}{\rho^{7.907}\cdot \gamma^{0.181}\cdot h^{0.249}}}\cdot \rho^{-8.907}\cdot \gamma^{-0.181}\cdot h^{-0.249}\\ -10.034\rho^{-11.034}\cdot \gamma^{-0.217}\cdot h^{-0.212} \end{array}\right|$$

(15) $$\left|\frac{dq}{d\gamma }\right|=\left|\begin{array}{l} -0.081\rho^{-5.193}\cdot \gamma^{-1.081}\cdot h^{-0.144}\cdot e^{-\frac{t}{\rho^{7.907}\cdot \gamma^{0.181}\cdot h^{0.249}}}-0.181\rho^{-5.193}\cdot \gamma^{-0.081}\cdot h^{-0.144}\cdot e^{-\frac{t}{\rho^{7.907}\cdot \gamma^{0.181}\cdot h^{0.249}}}\cdot \rho^{-7.907}\cdot \gamma^{-1.181}\cdot h^{-0.249}\\ -0.217\rho^{-10.034}\cdot \gamma^{-1.217}\cdot h^{-0.212} \end{array}\right|$$

(16) $$\left|\frac{dq}{dh}\right|=\left|\begin{array}{l} -0.144\rho^{-5.193}\cdot \gamma^{-0.081}\cdot h^{-1.144}\cdot e^{-\frac{t}{\rho^{7.907}\cdot \gamma^{0.181}\cdot h^{0.249}}}-0.249\rho^{-5.193}\cdot \gamma^{-0.081}\cdot h^{-0.144}\cdot e^{-\frac{t}{\rho^{7.907}\cdot \gamma^{0.181}\cdot h^{0.249}}}\cdot \rho^{-7.907}\cdot \gamma^{-1.181}\cdot h^{-1.249}\\ -0.212\rho^{-10.034}\cdot \gamma^{-0.217}\cdot h^{-1.212} \end{array}\right|$$

**Figure 7:**
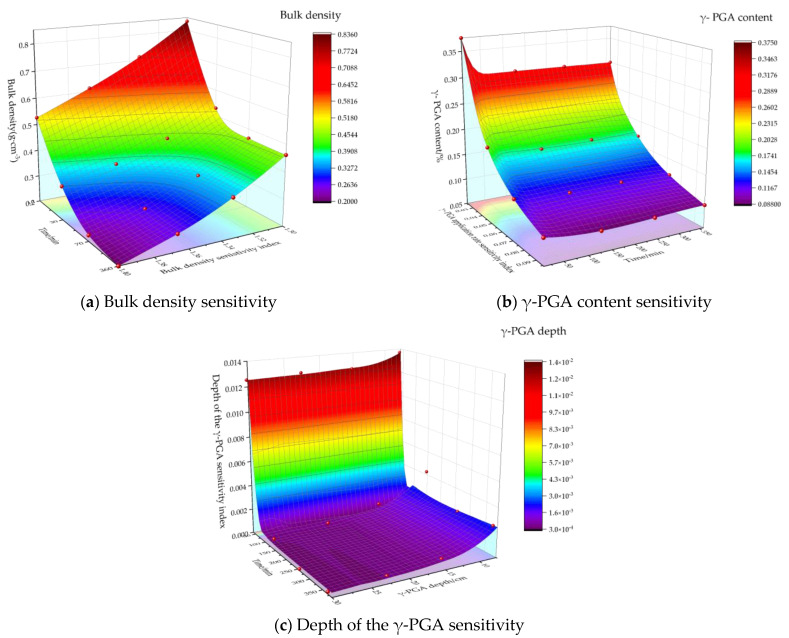
Sensitivity of various factors.

Taking the treatment of NO. 4 as an example, the sensitivity index of each influencing factor to soil infiltration rate was calculated, and the corresponding change curve was plotted. The results are depicted in Figure 7.

It is apparent that the relationship between the sensitivity index and each influencing factor is negatively correlated. The impact of soil bulk density and γ-PGA content on infiltration rate is significantly greater than that of γ-PGA application depth. Specifically, the soil bulk density had the greatest influence on the infiltration rate, followed by the γ-PGA content sensitivity, with the γ-PGA application depth having the least influence.

#### 3.2.4. Relationship between Cumulative Infiltration Amount and Wetting Front Migration Distance

To explore the relationship between the cumulative infiltration amount and the wetting front migration distance, these variables were compared and analyzed through point plotting, and the trend was delineated using points.

**Figure 8:**
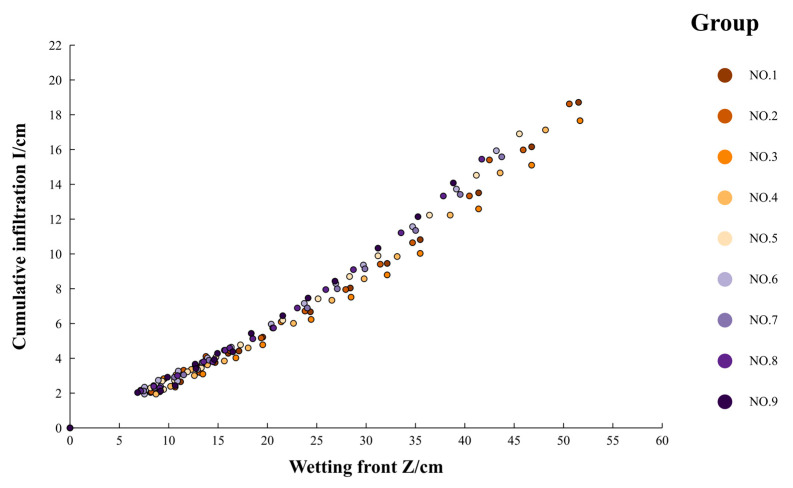
Comparison of cumulative infiltration and migration distance of wetting front.

From Figure 8, it is evident that the relationship between the cumulative infiltration amount and the wetting front migration distance closely resembles a linear trend. When considering the principle of water balance, this relationship can be specifically described by Formula (17):

(17) $$I=(\theta_{S}-\theta_{0})H=K_{1}H$$

In the formula, $K_{1}$ is the fitting coefficient, $\theta_{S}$ represents the saturated moisture content, cm^3^/cm^3^, and $\theta_{0}$ denotes the initial moisture content, cm^3^/cm^3^.

Subsequently, it was observed that the variation curve between the cumulative infiltration amount and the wetting front migration distance in Figure 7 aligns more closely with a power function relationship. This was incorporated into an amended version of Formula (17):

(18) $$I=(\theta_{S}-\theta_{0})H=K_{1}^{\prime }H^{\left(1+K_{2}^{\prime }\right)}$$

In the formula, $K_{1}^{\prime }$ is the fitting coefficient and $K_{2}^{\prime }$ is the modified exponent of the fit.

To simplify calculations, Formula (18) can be linearized by taking the logarithm of both sides. The parameters $K_{1}^{\prime }$ and $K_{2}^{\prime }$ can be determined through linear fitting lnI~lnH, then Formula (18) is transformed into the following:

(19) $$\ln I=\ln K_{1}^{\prime }+(1+K_{2}^{\prime })\ln H$$

The goodness of fit is compared between the conventional fitting equation and the modified fitting equation, as shown in Table 6.

Table 6 indicates that the coefficient of determination (*R*^2^) for the modified fitting equation exceeds 0.996, and the root mean square error (*ν_RMSE_*) has decreased from 0.55–1.10 to below 0.06. The average determination coefficient prior to modification improved by 2.2%, and the RMSE reduced by 93.1%. This suggests that compared to the original equation (Formula (12)), the modified equation (Formula (13)) provides a more precise and quantitative representation of the relationship between cumulative infiltration and wetting front migration distance, and is thus deemed reasonable.

**Figure 9:**
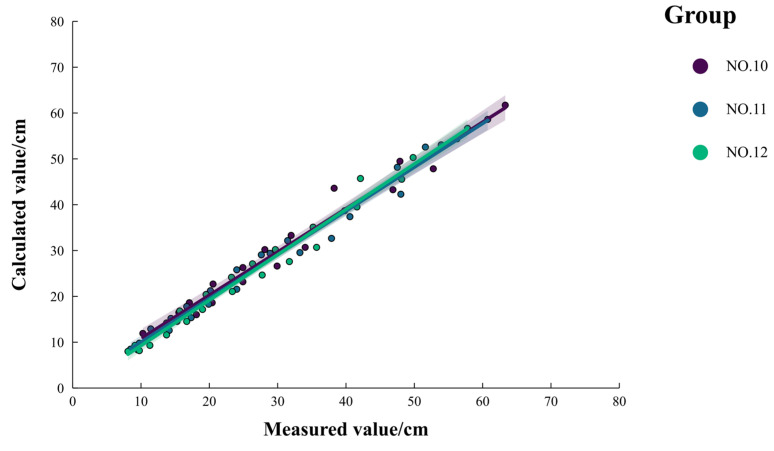
Comparison results of measured and calculated values of wetting front.

Using the data from sample NO. 10, NO. 11, and NO. 12, the reliability of the fitting model (Formula (13)) was verified, as depicted in Figure 9. The relative deviations between the predicted and measured wetting front migration distances were all less than 10%, indicating that the fitting model (Formula (13)) effectively captures the correlation between the wetting front migration distance and cumulative infiltration.

## 4. Discussion

(1) This study investigated the impact of γ-PGA on soil permeability, focusing exclusively on indoor experiments without field trials. The indoor experiment assessed variables such as soil substrate density, application rate, and the depth of γ-PGA application. Despite its confinement to indoor settings, this research lays the groundwork for subsequent field studies involving γ-PGA. A controlled application strategy for γ-PGA significantly improved the soil water content and permeability, thereby enhancing the water use efficiency in soil management practices. These results are crucial for improving crop growth conditions, reducing irrigation water usage, and advancing sustainable agriculture and ecological conservation. In this study, sandy loam soil was specifically selected. Future research should explore the effectiveness of γ-PGA across different soil types and crops, examining its long-term effects on soil biological activity and diversity, and verifying its practical benefits in agriculture through field trials. Effective agricultural strategies and policies are essential to maximize the use of scientific tools in agriculture, facilitating the dissemination of knowledge to farmers for a broader evaluation of its application potential and environmental impact.
(2) The findings indicate that γ-PGA significantly influences soil infiltration performance. However, the experiments were subject to various factors, leading to considerable discrepancies between the experimental and simulation results. Firstly, variables such as temperature, pH, radiation, and salinity at the input end may alter the stability of γ-PGA. Secondly, indoor experiments must consider fluctuations due to changes in conditions like air pressure, evapotranspiration, and freeze–thaw cycles. Lastly, the suitability of field experiments must account for factors such as rainfall, fertilization, and crop rotation methods in analyzing outcomes post-γ-PGA application. The current literature integrating these three aspects is limited, necessitating further investigation into the potential influences of γ-PGA in crop cultivation and the development of corresponding management strategies.
(3) In this study on soil permeability using γ-PGA, the composite of the van Genuchten and Brooks–Corey models was employed to enhance the accuracy of predicting soil hydraulic properties. However, the analysis was confined to gravelly soils [11]. It is noteworthy that the Barcelona Expansion Model (BExM), which improves upon the BBM modeling framework, has been extensively used to examine the properties of expansive materials [20,21]. Moving forward, the composite model utilized in this study could be integrated with the dual pore model. The BExM model could then be employed to analyze the irrecoverable deformation of γ-PGA under water ingress conditions. This approach would facilitate the development of macroscopic and microscopic pore structure models and the analysis of interactions between these dual pore structures. Such advancements will provide theoretical support for enhancing the accuracy of γ-PGA infiltration studies and their applicability to various soils.
(4) This study evaluated the effects of γ-PGA under controlled laboratory conditions, focusing on specific soil bulk densities (1.30 g/cm^3^, 1.35 g/cm^3^, and 1.40 g/cm^3^). However, the variability of soil bulk density under field conditions necessitates further exploration of its impact on γ-PGA efficacy. An increase in soil bulk density typically reduces soil porosity and increases compaction, potentially limiting the dispersion and water absorption capabilities of γ-PGA, thus affecting its water retention and infiltration properties [7,22]. Conversely, lower soil bulk density increases soil porosity and permeability, enhancing the water retention effect of γ-PGA. However, this also accelerates water infiltration and loss, potentially diminishing its long-term water retention capacity [14]. Moreover, optimizing soil bulk density may require considering specific soil types, crop requirements, and climatic conditions to achieve optimal γ-PGA application [8]. Future research should explore a broader range of soil bulk densities and incorporate field trials to validate the applicability and generalizability of laboratory findings.
(5) The study of the effects of γ-polyglutamic acid (γ-PGA) on soil hydrodynamic properties revealed that variations in γ-PGA application depth significantly impact soil moisture distribution and permeability. This impact is evident in the root zone interactions during crop cultivation, where the shallow application of γ-PGA can effectively retain moisture in the root zone, thereby improving water use efficiency [1,4]. Additionally, increased γ-PGA concentration leads to a decrease in the n-value, indicating a reduced water loss rate, which is crucial for irrigation management in arid and semi-arid regions, helping to minimize water loss and reduce irrigation frequency and water usage [3,6]. Furthermore, the application of γ-PGA can enhance soil water retention capacity and the efficiency of nutrient utilization such as nitrogen, phosphorus, and potassium, thereby increasing crop yields and reducing irrigation water consumption, offering significant agricultural benefits [2,6]. The polypeptide structure of γ-PGA interacts with soil particles, altering soil physical properties and enhancing moisture retention [23]. Its negative charge helps bind positively charged ions in the soil, thus influencing soil structure and porosity [2,4]. However, further field trials and long-term observational studies are needed to fully understand the effects of γ-PGA under different soil types and climatic conditions in order to develop more precise agricultural management strategies [13,14].
(6) Although Section 3.2.2 successfully quantified the nonlinear and linear phases of soil moisture infiltration using the parameters *A*, *B*, and *C*, and Section 3.2.3 further derived an expression for calculating infiltration rate, these analyses provide critical quantitative insights into the soil infiltration process. However, the study has yet to conduct a specific quantitative analysis of key physical parameters such as depth *h*, saturated water content *θs*, residual water content *θr*, bulk density *ρ*, and solution concentration. These factors play a crucial role in soil moisture transport and infiltration. Quantifying them is essential for more accurately describing soil hydrodynamics and optimizing irrigation strategies. Therefore, future research should focus on the mechanisms by which these variables influence infiltration, and develop mathematical models based on experimental and simulation data to enhance the scientific and effective management of soil moisture and agricultural water resources.

## 5. Conclusions

Through a combination of laboratory and simulation tests, this study established and verified the influence of varying application rates of γ-polyglutamic acid (γ-PGA) on soil moisture characteristic parameters. Additionally, the effects of soil bulk density, γ-PGA application depth, and γ-PGA content on soil infiltration characteristics and water-holding capacity were analyzed. The specific results are as follows:

(1) A comparison of the measured soil moisture parameters with the optimized inversion values under varying bulk densities and application rates of γ-PGA revealed that the coefficient of determination (*R*^2^) for the predicted soil moisture parameters exceeded 0.993. The RMSE values ranged from 0.001 to 0.024, MAE values from 0.001 to 0.017, and ME values from −0.0025 to 0.0019, all of which are close to zero. These findings indicate that the Hydrus-1D inversion, incorporating γ-PGA, yields more accurate soil moisture parameters. The application of γ-PGA did not significantly affect the retained water content but increased the upper limit of the saturated water content, thereby enhancing soil water-holding capacity. Furthermore, increased γ-PGA application facilitated earlier water loss but reduced the rate of water loss.
(2) Significant analysis showed that soil capacity (*ρ*) and γ-PGA application methods are crucial for the effective use of γ-PGA. Reduced soil capacity and deeper γ-PGA application enhance soil cumulative infiltration. The cumulative infiltration model, determined by an analog capacitance charging model, effectively describes the quantitative relationship between cumulative infiltration, the migration distance of the wetting front, various influencing factors, and infiltration time.
(3) The proposed model identifies a power index function relationship between the three fitted parameters—infiltration volume constant A, time constant B, and stable permeability C—and soil bulk density, γ-PGA application depth, and γ-PGA content. The correlation analysis reveals that different factors uniquely influence these parameters: a reduction in the bulk density ρ significantly enhances the water storage capacity in soil capillary pores (positively correlated with volume constant A, *p* < 0.01), yet substantially decreases the water retention time during the early stages of infiltration (negatively correlated with the time constant B, *p* < 0.01), and improves the water infiltration rate during the stable infiltration stage (positively correlated with the stable infiltration rate C, *p* < 0.01). Additionally, an increase in γ-PGA application rate effectively boosts the water storage capacity in soil capillary pores and reduces the retention time of water early in infiltration, but does not affect the infiltration rate in the stable stage. Conversely, an increase in the γ-PGA application depth negatively impacts the water storage capacity in soil capillary pores and shortens the transition from nonlinear to linear water infiltration, yet it markedly enhances the stable infiltration rate.
(4) A sensitivity analysis was used to determine the impact of each factor on soil infiltration rate. It was found that the influence of soil bulk density and γ-PGA content on infiltration rate is significantly greater than that of γ-PGA application depth. Among these, soil bulk density had the most substantial impact on infiltration rate, followed by γ-PGA application rate sensitivity, with γ-PGA application depth having the least impact.
(5) The relationship between cumulative infiltration amount and wetting front migration distance conforms more closely to a power function, with the coefficient of determination (*R*^2^) of the modified fitting equation exceeding 0.996. The root mean square error (*ν_RMSE_*) was also reduced from 0.55–1.10 to less than 0.06, an increase of 2.2% over the average determination coefficient of the pre-correction fitting equation, and a 93.1% reduction in RMSE. Reliability verification further demonstrates that the revised calculation model more accurately describes the correlation between wetting front migration distance and cumulative infiltration amount.

## Figures and Tables

**Table 1:** The particle analysis of the tested soil.

Soil Type	Particle Gradation Composition			Soil Physical and Chemical Parameters				
	Volume Fraction/%			Initial Moisture (%)	Saturated Moisture (%)	Initial Nitrate Nitrogen (mg·kg^−1^)	Initial Ammonium Nitrogen (mg·kg^−1^)	PH Value (-)
	Clay (<0.002 mm)	Silt (≥0.002~0.02 mm)	Sand (≥0.02~2 mm)					
Sandy loam	2.30	10.50	87.20	5.30	38.59	8.98	16.54	9.15

**Table 2:** Design of the orthogonal test.

Treatment	Bulk Density	Depth	Content of γ-PGA
	(g/cm^3^)	(cm)	(%)
NO. 1	1.30	5~25	0.10
NO. 2	1.30	15~35	0.20
NO. 3	1.30	25~45	0.30
NO. 4	1.35	5~25	0.20
NO. 5	1.35	15~35	0.30
NO. 6	1.35	25~45	0.10
NO. 7	1.40	5~25	0.30
NO. 8	1.40	15~35	0.20
NO. 9	1.40	25~45	0.10
NO. 10	1.32	8~28	0.12
NO. 11	1.34	13~33	0.19
NO. 12	1.38	18~38	0.28

**Table 3:** Soil hydraulic parameters of soils treated with different γ-PGA application rates.

Content of γ-PGA(%)	Model Parameters					Goodness of Fit				
	*ρ*	*θr*	*θs*	α		*Ks*	*R* ^2^	*ν_RMSE_*	*ν_MAE_*	*ν_ME_*
	Bulk Density (g/cm^3^)	Residual Moisture	Saturated Moisture (cm^3^/cm^3^)	(1/cm)	n (-)	Saturated Hydraulic Conductivity (cm/min)				
0	1.3	0.0308	0.4	0.0399	1.46	0.095	0.999	0.004	0.003	0.0005
0	1.35	0.031	0.3916	0.0369	1.48	0.085	0.996	0.019	0.017	−0.0008
0	1.4	0.0307	0.3806	0.0361	1.49	0.073	0.999	0.002	0.002	−0.0004
0.1	1.3	0.031	0.3862	0.038	1.43	0.092	0.999	0.003	0.002	0.0019
0.2	1.3	0.0328	0.4271	0.0395	1.4	0.089	0.993	0.024	0.017	0.0003
0.3	1.3	0.033	0.4293	0.0407	1.36	0.084	0.999	0.004	0.003	−0.0025
0.1	1.35	0.0315	0.4045	0.0372	1.45	0.08	0.997	0.018	0.012	0.001
0.2	1.35	0.0318	0.4186	0.0381	1.43	0.078	0.999	0.006	0.004	0.0001
0.3	1.35	0.032	0.4202	0.039	1.4	0.074	0.999	0.002	0.002	0.0002
0.1	1.4	0.0308	0.3964	0.0366	1.47	0.071	0.999	0.002	0.002	−0.0003
0.2	1.4	0.0309	0.4117	0.037	1.43	0.069	0.999	0.002	0.002	−0.0001
0.3	1.4	0.031	0.4216	0.0376	1.41	0.067	0.999	0.001	0.001	−0.0001

**Table 4:** Influence of different factors in different levels of the cumulative infiltration.

Level	*ρ*	h	γ
	Bulk Density	Depth	Content of γ-PGA
1	18.23 Aa	15.18 Aa	16.24 Aa
2	16.00 Bb	16.44 Bb	15.99 Ba
3	13.86 Cc	16.47 Cb	15.87 Ba

Note: different lowercase letters indicate significant differences (*p* < 0.05), while different uppercase letters indicate extremely significant differences (*p* < 0.01).

**Table 5:** Fitting parameters of NO. 1–NO. 9 cumulative infiltration.

Treatment	$I=A\cdot (1-e^{-\frac{t}{B}})+C\cdot t$		
	Analog Values		
	Volume Constant A	Time Constant B	Stable Infiltration Stage C
NO. 1	2.590	15.190	0.044
NO. 2	2.640	15.497	0.044
NO. 3	2.632	14.778	0.042
NO. 4	2.528	15.750	0.041
NO. 5	2.613	16.552	0.040
NO. 6	2.605	15.489	0.037
NO. 7	2.447	16.500	0.036
NO. 8	2.500	17.116	0.036
NO. 9	2.560	16.213	0.032

**Table 6:** Comparison table of fitting equation parameters.

Treatment	$I=K_{1}H$			$I=K_{1}^{\prime }H^{\left(1+K_{2}^{\prime }\right)}$			
	*K*_1_	*R* ^2^	*ν_RMSE_*	$K_{1}^{\prime }$	$K_{2}^{\prime }$	*R* ^2^	*ν_RMSE_*
NO. 1	0.319	0.985	1.052	0.170	0.166	0.997	0.054
NO. 2	0.322	0.988	1.014	0.174	0.164	0.998	0.053
NO. 3	0.305	0.986	0.923	0.172	0.153	0.998	0.054
NO. 4	0.316	0.986	0.911	0.182	0.147	0.997	0.053
NO. 5	0.330	0.987	0.852	0.195	0.144	0.998	0.049
NO. 6	0.331	0.989	0.736	0.209	0.128	0.998	0.051
NO. 7	0.317	0.988	0.764	0.200	0.125	0.999	0.048
NO. 8	0.330	0.989	0.732	0.211	0.124	0.998	0.049
NO. 9	0.329	0.992	0.552	0.233	0.100	0.996	0.060

## Data Availability

The original contributions presented in the study are included in the article, further inquiries can be directed to the corresponding author/s.
